# Pre- and Post-Surgical Dysphagia in Adults with Tumors of the Posterior Fossa: A Prospective Blinded Study

**DOI:** 10.3390/cancers12092561

**Published:** 2020-09-09

**Authors:** Sriramya Lapa, Johanna Quick-Weller, Christiane Nasari, Rainer Dziewas, Florian Gessler, Marlies Wagner, Tobias Warnecke, Elke Hattingen, Volker Seifert, Jürgen Konczalla

**Affiliations:** 1 Department of Neurology, University Hospital Frankfurt, 60528 Frankfurt, Germany; christiane.nasari@kgu.de; 2 Department of Neurosurgery, University Hospital Frankfurt, 60528 Frankfurt, Germany; Johanna.Quick@kgu.de (J.Q.-W.); florian.gessler@kgu.de (F.G.); Volker.Seifert@kgu.de (V.S.); juergen.konczalla@kgu.de (J.K.); 3 Department of Neurology, University Hospital Münster, 48149 Münster, Germany; Rainer.Dziewas@ukmuenster.de (R.D.); tobias.warnecke@ukmuenster.de (T.W.); 4 Department of Neuroradiology, University Hospital Frankfurt, 60528 Frankfurt, Germany; marlieswa@gmx.de (M.W.); elke.hattingen@kgu.de (E.H.)

**Keywords:** swallowing, dysphagia, posterior fossa tumor, brain tumor, aspiration

## Abstract

**Simple Summary:**

Dysphagia is a known complication of posterior fossa tumor resection, but data regarding risk factors and clinical course are sparse, in particular in adults. The purpose of this study was to investigate frequency, severity and outcome of swallowing disorders in adults undergoing PFT-surgery to improve presurgical counseling and postsurgical therapeutic care. Our findings demonstrate that dysphagia is a frequent finding before and after surgery putting patients at risk for aspiration and pneumonia. We provide clinical predictors which might be helpful in identifying dysphagic patients in order to determine the safest feeding route. This might lead to both improvement of outcome and reduction of medical complication.

**Abstract:**

Background: While swallowing disorders are frequent sequela following posterior fossa tumor (PFT) surgery in children, data on dysphagia frequency, severity, and outcome in adults are lacking. The aim of this study was to investigate dysphagia before and after surgical removal of PFT. Additionally, we tried to identify clinical predictors for postsurgical swallowing disorders. Furthermore, this study explored the three-month outcome of dysphagic patients. Methods: In a cohort of patients undergoing PFT surgery, dysphagia was prospectively assessed pre- and postoperatively using fiberoptic endoscopic evaluation of swallowing. Patients with severe dysphagia at discharge were re-evaluated after three months. Additionally, clinical and imaging data were collected to identify predictors for post-surgical dysphagia. Results: We included 26 patients of whom 15 had pre-operative swallowing disorders. After surgery, worsening of pre-existing dysphagia could be noticed in 7 patients whereas improvement was observed in 2 and full recovery in 3 subjects. New-onset dysphagia after surgery occurred in a minority of 3 cases. Postoperatively, 47% of dysphagic patients required nasogastric tube feeding. Re-evaluation after three months of follow-up revealed that all dysphagic patients had returned to full oral intake. Conclusion: Dysphagia is a frequent finding in patients with PFT already before surgery. Surgical intervention can infer a deterioration of impaired swallowing function placing affected patients at temporary risk for aspiration. In contrast, surgery can also accomplish beneficial results resulting in both improvement and full recovery. Overall, our findings show the need of early dysphagia assessment to define the safest feeding route for the patient.

## 1. Introduction

The posterior fossa is part of the cranial cavity, which includes the brain regions and cranial nerves (CNs) below the tentorium. Clinical presentation associated with posterior fossa tumors (PFTs) usually comprises increased intracranial pressure, focal neurological deficits secondary to compromise of the brain stem, and cranial nerves or cerebellar tissue [1]. As those structures also play a critical role in the precise and efficient execution of swallowing, and dysphagia might occur as a clinical manifestation of the tumor itself or a possible sequel after surgical removal [2,3,4,5]. The presence of dysphagia may impair the initiation, coordination, or maintenance of swallowing, placing the patient at risk of aspiration. Aspiration is the most clinically significant symptom of dysphagia that is strongly associated with pneumonia, malnutrition, and increased mortality and morbidity [4,6,7,8]. Dysphagic patients also have a higher likelihood to undergo tracheostomy or gastrostomy tube placement [9]. Early identification of patients at risk for dysphagia enables timely diagnostic and therapeutic care to prevent aspiration pneumonia and further respiratory complications and could therefore improve patient outcomes [6,10]. Data on dysphagia in PFT is very rare. Most studies focused on pediatric populations and recorded a frequency of dysphagia ranging from 33% to about 70% [4,11,12,13]. The only data in adults is from Wadhwa et al. reporting on 29% of dysphagic patients in their cohort [1]. Most of the underlying studies have the methodical concerns of a retrospective design, varying time of assessment, and profession. In addition, most data rely on the clinical swallowing examination, which, in contrast to instrumental diagnostic (e.g., fiberoptic endoscopic evaluation of swallowing, videofluoroscopy), suffers from low sensitivity and specificity in detecting dysphagia and dysphagia severity [14,15,16].

To our knowledge, there is no detailed and systematic prospective investigation on dysphagia and dysphagia outcome using instrumental swallowing assessment in adult patients prior to and after PFT surgery. The aim of this study was to provide data on expected prevalence, clinical predictors, and prognostic indicators of dysphagia and aspiration. This may help to streamline resources and to speed up professional dysphagia assessment by speech and language pathologists (SLPs). In addition, the provision of more data on the presentation and outcome of swallowing disorders will facilitate pre-surgical counseling for patients affected by PFT.

## 2. Results

### 2.1. Patient Characteristics

Twenty-six patients (47% female) with PFT were enrolled in this study. The mean age was 49 ± 14 years and the mean size of the tumor was 36 ± 10mm (range 20–53). Brainstem compression was radiologically confirmed in 65% of cases; in 77%, the tumor was adjacent to cranial nerves. Total surgical removal of the tumor was possible in 11/26 (42%) of cases. Mean time of intubation was 11.5 ± 19.2 h (range 4.5–104 h) and surgery lasted for 4.1 ± 1.6 h (range 2.1–8 h).

Histopathology confirmed meningioma WHO grade I in 23% of the patients, meningioma WHO grade II in 12%, vestibular schwannoma in 23%, metastases in 19%, and tumors of other entities in the remaining 23%.

Pneumonia after surgery was present in one patient. Dysphagic patients had a longer intensive care unit stay (*p* = 0.02) whereas no difference between dysphagic and non-dysphagic patients concerning length of overall hospital stay could be observed. Baseline data and clinical variables of the study population are displayed in Table 1. 

### 2.2. Pre-Surgical Dysphagia Assessment

Paresis of one or more cranial nerves involved in swallowing was objectified in 6/26 (23%) patients (V *n* = 2, VII *n* = 5, IX *n* = 2, X *n* = 1, XII *n* = 2). Dysarthria and dysphonia were present in 2/26 (8%) cases. In general, the pre-operative tumor size was moderately associated with dysphagia severity (r = 0.42; *p* = 0.03).

According to fiberoptic endoscopic evaluation of swallowing (FEES), 15/26 (58%) patients were classified as having pre-surgical (pre-OP) dysphagia, of whom 10 (10/15; 67%) denied swallowing problems. Presence of pre-OP dysphagia was significantly associated with post-surgical (post-OP) dysphagia (OR: 17.3 *p* = 0.005). The most common FEES findings in the dysphagic population were premature spillage (in 14/15; 93%) and pharyngeal residues (in 14/15; 93%), respectively. Penetration into the laryngeal vestibule was predeglutitive in all cases and occurred in 27% (4/15) while aspiration was observed only once (6%).

### 2.3. Post-Surgical Dysphagia Assessment

After surgery, 14/26 (54%) patients suffered from paresis of one or more cranial nerves involved in swallowing (V *n* = 4, VII *n* = 11, IX *n* = 7, X *n* = 6, XII *n* = 3). Post-OP dysarthria was present in 9/26 (34%) and post-OP dysphonia in 4/26 (15.4%) subjects. Abnormal gag reflex and wet-voice could be objectified in 3/26 patients (11.5%), and abnormal volitional cough and cough after swallow were detected in 4/26 (15.4%) and 6/26 (23.1%), respectively (Table 2).

Fifteen (58%) patients had post-OP dysphagia, with 7/15 (47%) suffering from severe swallowing disorders (fiberoptic endoscopic dysphagia severity scale (FEDSS) 4–6) necessitating nasogastric tube (NGT) feeding and nil per os. In 7/15 (46.7%) patients, pre-OP dysphagia worsened whereas improvement of pre-existing dysphagia could be observed in 2 (13.3%) and full recovery in 3 (20%) patients.

In 3 (20%) subjects, pre-op dysphagia remained unchanged after surgery. New onset of dysphagia after surgery occurred in the remaining 3 cases (20%). The average of improvement and deterioration was 3 points according to the FEDSS, respectively. However, the extent of residues significantly increased compared to pre-surgical evaluation.

In analogy to the pre-OP evaluation, premature spillage and pharyngeal residues were the most common findings in 87% (13/15), respectively. Penetration and aspiration could be observed more frequently (penetration, 78% vs. 27%, aspiration, 14% vs. 6%) after surgery.

While handling of saliva was not affected in any patient before surgery, 47% (7/15) suffered from mild to moderate pooling of saliva in the piriform sinus and/or vallecular in the post-OP evaluation.

### 2.4. Predictors for Post-Surgical Dysphagia

Brainstem compression (*p* = 0.05) and post-OP dysarthria (*p* = 0.0038) were identified to be significant predictors for post-OP dysphagia. Paresis of CN IX (*p* < 0.001) and CN X (*p* = 0.03) as well as abnormal gag reflex (*p* = 0.013), abnormal volitional cough (*p* = 0.002), wet-voice (*p* = 0.013), and cough after swallow (*p* = 0.002) showed a significant association with severe dysphagia defined by an FEDSS of 4–6 (Table 3).

Dysphagia severity correlated with post-OP paresis of CN IX (*p* = 0.002) and CN X (*p* = 0.001) and post-OP dysarthria (*p* < 0.001) and dysphonia (*p* = 0.02). Additionally, abnormal gag reflex (*p* = 0.034), abnormal volitional cough (*p* = 0.006), wet-voice (*p* = 0.011), and cough after swallow (*p* = 0.001) showed a significant association with dysphagia severity. Additionally, tumors with brainstem compression were more likely to be associated with worse swallowing functions (*p* = 0.029).

After adjusting for multiple testing, only post-OP dysarthria remained as a predictor for post-OP dysphagia (Table 2). For severe dysphagia, paresis of CN IX, abnormal cough, and cough after swallow all post-OP persisted as significant predictors (Table 3). Additionally, a higher FEDSS was significantly associated with the occurrence of post-OP CN paresis IX and CN X and dysarthria.

Using receiver operating characteristics (ROC) analysis, a cut off of 6.5 h of intubation allowed for the differentiation between severely dysphagic and non-dysphagic patients (AUC 0.847 (95% CI, 0.697–0.997)) with a sensitivity and specificity of 81.3% and 80%, respectively. Concerning the duration of surgery, a cut off of 3.25 h was identified with a sensitivity of 81.3% and specificity of 80% (AUC 0.859 [95% CI 0.711–1.000) to distinguish between patients suffering from severe dysphagia and no dysphagia.

### 2.5. Follow Up and Outcome of Post-Surgical Dysphagia

During hospitalization, dysphagia fully recovered in 60% (9/15) of participants whereas 27% (4/15) returned to oral intake but still needed special preparation and/or had to avoid specific textures. Two patients (2/15) still depended on nasogastric tube (NGT) feeding at discharge and received percutaneous endoscopic gastrostomy (PEG) placement during rehabilitation. Tracheotomy was performed in one case due to a failure to wean and inability to handle excessive secretions.

Three-month follow-up of patients with severe dysphagia at discharge revealed persistent mild dysphagic symptoms like premature spillage and mild residues. All patients had returned to a full oral diet. Additionally, all PEG and/or tracheostomy tubes could be removed.

### 2.6. Interrater Reliability

The interrater reliability for the assessment of dysphagia pre-OP was substantial, with a k-value of 0.69 (*p* < 0.001), and good for the pre-OP FEDSS, with a k-value of 0.76 (*p* < 0.001). The interrater reliability was perfect to almost perfect, with a k-value of 1 for the evaluation of post-OP dysphagia and a k-value of 0.90 for the post-OP FEDSS, respectively.

## 3. Discussion

In the present study, we evaluated the prevalence and outcome of patients with dysphagia before and after surgical resection of PFT. One of our main findings was the identification of pre-OP dysphagia in as many as 60% of patients, with two thirds of affected subjects not being aware of it. This finding is in contrast to previous studies, which reported either no or significantly lower rates (as low as 6.5%) of pre-existing dysphagia [1,4]. However, data must be interpreted with caution as, at least partly, they were collected in pediatric populations, limiting comparability. An additional reason for this difference might be our stringent use of FEES, which is known to offer an excellent diagnostic sensitivity in the evaluation of swallowing function. Further, clinical swallowing assessment and/or relying on medical history are prone to missing relevant pre-OP swallowing dysfunction [15,17].

The incidence of dysphagia did not increase after surgery. Worsening of pre-existing swallowing function could be observed in about half of the cohort to a degree necessitating nasogastric tube feeding (NGT). New onset of dysphagia after surgery occurred only in a minority of three cases. In light of these findings, it can be assumed that the tumor itself affects swallowing physiology [10,18,19]. Surgical intervention of the tumor located in brain areas relevant for swallowing leads to deterioration of the already impaired system. Otherwise, surgical approaches can also accomplish beneficial results possibly due to reduction of focal compression and/or increased intracranial pressure as witnessed in about 25% of our dysphagic subjects.

To understand why both improvement and deterioration is observed after surgical intervention, further analyses, including tumor pathology, tumor location, presence and treatment of hydrocephalus, and extent of resection, are warranted.

This study is the first to provide detailed insight into the development of post-OP dysphagia in adults undergoing PFT surgery. In contrast to conventional assumptions, post-OP dysphagia is most commonly not only a result of surgical intervention [1,4]. Additionally, the documented improvement of pre-existing dysphagia through surgical intervention needs to be highlighted as a novel finding.

The importance of pre-OP dysphagia for post-surgical swallowing complications is supported by its role as a significant risk factor for post-OP dysphagia. Furthermore, the presence of post-OP dysarthria was identified as a valuable parameter in distinguishing dysphagic from non-dysphagic patients. Additionally, both the duration of intubation (>6.5 h) and surgery (>3 h) were significantly correlated with post-OP dysphagia, with a sensitivity and specificity of 80%, respectively. These parameters might prove useful in clinical decision pathways to risk-stratify identify patients in need of a thorough swallowing assessment by an SLP after surgery.

Focusing on severe dysphagia with dependence of NGT feeding, abnormal gag reflex, abnormal volitional cough, as well as both voice change and cough after swallow were found to have predictive potential. Additionally, paresis of CN IX and X were more likely associated with severely impaired swallowing function and increased dysphagia severity. These findings are in accordance with previous studies on dysphagia and PFT describing severe swallowing disturbance resulting from combined injury to cranial nerves IX, X, and XII [4,13,20,21]. Consequently, hypopharyngeal residues with insufficient bolus clearance and subsequently penetration were prominent findings during FEES in our cohort. Additionally, premature spillage as a result of delayed pharyngeal swallow could be observed just as often. Post-surgical brainstem edema and/or brainstem disturbance impacting the forward signal from the swallowing centers to cranial nerves might be discussed as a potential underlying cause of the latter finding [7].

Based on the FEES findings, we initiated temporary NGT feeding in about 50% of patients while the remaining dysphagic patients continued oral intake with special preparation and/or avoiding special food consistencies. Possibly, the early screening for dysphagia and the stringent use of FEES to determine nutrition and therapeutic management might explain the low rate of post-OP pneumonia (*n* = 1) in our cohort. In contrast to previous studies, we also did not observe any significant differences regarding the length of hospital stay between dysphagic and non-dysphagic patients [4,7,11,22,23].

We provide valuable clinical predictors for early identification of patients at risk of severe post-OP dysphagia. This may lead to the rapid introduction of further instrumental swallowing assessment for establishing the best means of a feeding route and to determine patient-tailored interventions for swallowing rehabilitation. Although most studies investigating dysphagia in PFT used VFS to assess swallowing function, FEES has proven its value as a readily applicable bedside diagnostic tool, which also enables the assessment of intensive care unit (ICU) patients. Furthermore, application of the FEDSS, which was originally conceived for stroke, proved useful in quantifying dysphagia severity in PFT patients (due to the similarity of dysphagia patterns in patients with infratentorial brain tumor lesions and brainstem strokes) [24]. The FEDSS additionally offers nutritional recommendations and is directly linked to appropriate protective and rehabilitative measures.

While data on the outcome of patients suffering from post-OP dysphagia after PFT surgery is lacking, our study provides first evidence concerning recovery from swallowing disorders. During hospitalization, post-OP dysphagia recovered in approximately 60% of affected subjects; only two patients required NGT-feeding at discharge. In the course of follow-up, placed tracheostomy tubes and PEGs could be removed and all dysphagic patients returned to full oral intake. The three-month follow-up FEES examination showed mild dysphagic symptoms like premature spillage and mild residues.

Thus, it can be assumed that new onset and/worsening of pre-existing dysphagia after surgery is commonly a temporary symptom probably resulting from manipulation of the CN and/or brainstem edema.

Limitations of this study include its small sample size, thus limiting its statistical power. Since adult PFT are rare, tumor entities, localization, and surgical approaches are heterogeneous. Hence, a detailed investigation of the influence of these factors was not feasible in this pilot study. Additionally, we neither performed a dedicated analysis of brain magnetic resonance imaging nor are tractography data available, thus limiting insight into additional factors leading to postsurgical dysphagia beside from surgical intervention. Future studies, including techniques like, e.g., diffusion tensor imaging (DTI), are warranted to address this issue in order to fully understand the nature of dysphagia following posterior fossa surgery [25,26,27].

However, this study features one of the largest adult PFT cohorts so far. Further strengths are the prospective design and a thorough investigation of speech, voice, swallowing, and cranial nerve functions before and after surgery. Moreover, we stringently used instrumental diagnostics (FEES) to evaluate the presence of dysphagia and dysphagia severity.

## 4. Material and Methods

### 4.1. Patients

Between January 2015 and December 2017, consecutive patients who presented to the Department of Neurosurgery of the Goethe University Hospital in Frankfurt, Germany, for surgical treatment of posterior fossa tumor were recruited for this study. Inclusion criteria were age > 18 years and surgical removal of PFT. Exclusion criteria comprised a known history of other concomitant disease likely to cause dysphagia (e.g., prior brainstem stroke, Parkinson disease).

The study was approved by the local ethics committee (ethic code: 305/13) of the Goethe University Hospital Frankfurt and was conducted according to the principles of the Declaration of Helsinki. Written and informed consent was obtained from all participants of the study prior to participation.

### 4.2. Dysphagia Assessment

All patients received a full assessment of speech, voice, and swallowing function including fiberoptic endoscopic evaluation of swallowing (FEES) before and after surgery. Post-surgical examination was performed 24 h post-extubation to avoid concomitant effects of sedation and/or intubation.

In detail, in a first step, paresis of cranial nerves involved in swallowing (CN V, CN VII, CN VIII, CN XII) as well as the presence of dysarthria, dysphonia, abnormal volitional cough, and abnormal gag reflex were evaluated. A simple water swallowing test was performed to assess cough and voice change after swallow [20,28,29]. Additionally, patients were asked for their medical history of swallowing problems.

In a second step, all patients underwent FEES to assess the presence of dysphagia and to classify dysphagia severity. Patients were rated as being dysphagic if one or more of the following signs of swallowing dysfunction were detected during FEES: Disturbed management of secretions (i.e., pooling or aspiration of saliva), penetration or aspiration of any food consistency, and relevant pharyngeal food residues after the swallow or premature spillage [30,31]. Dysphagia severity was rated according to the fiberoptic endoscopic dysphagia severity scale (FEDSS), with 1 scoring best and 6 being worst. Patients were categorized as mildly dysphagic (1–3) and severely dysphagic (4–6) [30,32]. Patients with severe dysphagia at discharge received both a clinical and instrumental (FEES) follow-up examination after three months.

FEES equipment consisted of a 3.1-mm-diameter flexible fiberoptic rhinolaryngoscope (ENF-P4, Olympus, Hamburg Germany), a 150W light source for endoscopic application (rp-150), a camera (rpCam62, S/N), a color monitor (7′-TFT-EIZO, 1500:1), and a video recorder (1/2″ CCD-Kamera, rp Cam62). All examinations were videotaped and saved on a server for later review. FEES procedures were performed by a team of two speech and language pathologists, both having several years of experience with this diagnostic tool.

A standardized FEES protocol was applied as previously described [32]. To assess vagal nerve paresis, patients were observed during respiration and phonation (e.g., /ah/). Patients presenting asymmetric vocal fold movement (reduced diadochokinesis, incomplete abduction or adduction) and/or glottis configuration (vocal fold bowing, thin vocal fold, shorter vocal fold) were judged as having vagal nerve paresis [21].

The swallowing examination started with studying the patient’s handling of his oropharyngeal secretions. In case of saliva pooling with penetration and/or aspiration without protective reflex, severe dysphagia was suspected, and the examination was cancelled. Patients who were able to handle their saliva without penetration or aspiration received semisolid (applesauce) consistency followed by liquids and solid food (white bread). All food was dyed with blue food coloring for better contrast with pharyngeal and laryngeal mucosa. Each consistency was regularly tested 3 times.

All videos (pre-surgical, post-surgical, and after 3 months) were independently scored by two raters who were blinded to the patients and their clinical conditions. For final analysis of the results, disagreement concerning the presence of dysphagia and the severity of dysphagia in terms of the FEDSS was discussed until consensus was reached.

In addition, patients were evaluated for the occurrence of (aspiration) pneumonia and necessity of re-intubation during hospitalization [33]. Furthermore, the duration of intubation and surgery were captured.

Evaluation of the size and localization of the tumor as well as the presence of brainstem compression were evaluated by an experienced neuro-radiologist reviewing pre-surgical magnet-resonance imaging.

### 4.3. Statistical Analysis

Quantitative data are presented as median, range, and mean (±SD). The Wilcoxon signed rank test was used for paired comparisons of quantitative parameters. Spearman correlation was used to estimate linear relationships. Fisher’s exact test and logistic regression were used to predict binary outcomes. Statistical analyses were performed in R (version 3.4.4, R Core Team, 2018; www.r-project.org). All statistical tests were performed two-sided and a *p*-value < 0.05 was considered to indicate statistical significance. Bonferroni correction was performed to adjust for multiple testing. A cut-off value for the duration of surgery and intubation was determined via receiver operating characteristics (ROC) analysis.

## 5. Conclusions

In conclusion, our study provides evidence regarding the presence, characteristics, and predictors of dysphagia before and after PFT surgery. It highlights the need for pre-surgical counseling and post-surgical intervention by an SLP and further instrumental evaluation, in cases of clinical signs for severe dysphagia. Increased awareness and early introduction of the SLP may help to optimize patient management in order to reduce health risk, length of hospitalization, and increase quality of life.

## Figures and Tables

**Table 1:** Demographic, Surgical, and Clinical Data

N	Age	Sex	Histology	Localization	Tumor Size	Brainstem Compression	Extend of Resection	Duration of Surgery (h)	FEDSS Pre-OP	FEDSS Post OP
1	64	F	Metastasis (LCNEC)	Intraaxial paramedian	41	No	CR	2.2	-	-
2	59	F	Meningioma WHO grade II	Petroclival	41	Yes	IR	6.4	2	3
3	57	M	Meningioma WHO grade II	Petrous bone	20	No	CR	2.2	-	-
4	47	F	Meningioma WHO grade I	Petrous bone and Foramen magnum	36	Yes	IR	5.7	1	1
5	59	F	Meningioma WHO grade I	Tentorium	26	No	CR	3.1	-	-
6	48	F	Epidermoid tumor	Petroclival	38	Yes	ST	4.6	1	6
7	63	M	Metastasis (Lung Cancer)	Cerebellum	27	No	CR	4.1	1	4
8	27	M	Vestibular Schwannoma WHO grade I	CN VIII	33	Yes	IR	4.4	-	2
9	36	M	Vestibular Schwannoma WHO grade I	CN VIII	22	Yes	IR	3.5	-	-
10	41	F	Medulloblastoma WHO grade IV	Intraaxial paramedian	37	No	CR	3.1	5	-
11	65	M	Benign cyst	Median intraaxial cystic	36	No	CR	3.1	1	1
12	22	M	Schwannoma WHO grade I IX	CN IX u. X	26	Yes	ST	5.6	1	5
13	31	M	Pilocytic Astrocytoma WHO grade I	Median intraaxial zystisch	41	No	ST	4.2	-	-
14	74	F	Meningioma WHO grade I	Tentorium	45	No	CR	10.2	1	2
15	64	M	Metastasis (Lung cancer)	Cerebellumintraaxial	34	Yes	CR	2.5	-	-
16	52	M	Metastasis (Gastric Cancer)	Cerebellumintraaxial	24	Yes	CR	2.1	1	1
17	34	M	Epidermoid tumor	CPA and Cavum meckeli	38	Yes	ST	2.8	-	-
18	68	F	Metastasis (CRC)	Clivus	31	Yes	CR	2.6	1	-
19	32	M	Meningioma WHO grade I	Tentorium	52	Yes	IR	7.9	1	6
20	50	F	Meningioma WHO grade II	Tentorium	31	No	CR	2.5	-	-
21	49	F	Arachnoid Cyst	Petroclival	53	Yes	CR	3.1	4	-
22	53	F	Meningioma WHO grade I	CPA	45	Yes	IR	5	5	4
23	65	M	Vestibular Schwannoma WHO grade I	CPA	45	Yes	IR	4.2	1	5
24	50	F	Vestibular Schwannoma WHO grade I	CPA	21	Yes	ST	3.4	-	5
25	47	F	Meningioma WHO grade I	CPA and Cavum meckeli	46	Yes	CR	5.6	3	1
26	23	M	Vestibular Schwannoma WHO grade I	Intrameatal	53	Yes	CR	6.6	-	3

CPA = Cerebellopontine Angle, CR = Complete resection; CRC = Colorectal Cancer, F = Female; FEDSS = Fiberoptic Endoscopic Dysphagia Severity Scale; IR = Incomplete Resection; M = Male, LCNEC = Large Cell Neuroendocrine Carcinoma, ST = Subtotal.

**Table 2:** Predictors for Post-Surgical Dysphagia.

Clinical Parameters	All	No Dysphagia	Dysphagia	*p*-Value
***n* (%)**	26	11 (42.3)	15 (60)	-
Age, years (SD) ^a^	49 (±15)	50 (±13)	48 (±16)	0.84
Tumor size, mm (SD) ^a^	36 (±10)	34 (±10)	37 (±10)	0.42
Compression of brainstem, *n* (%) ^b^	17 (65)	5 (19.2)	12 (46.2)	0.046 *
Post-OP Paresis V, *n* (%) ^b^	4 (15.38)	1 (3.8)	3 (11.5)	1
Post-OP Paresis VII, *n* (%) ^b^	11 (42.3)	3 (11.5)	8 (30.8)	0.11
Post-OP Paresis IX, *n* (%) ^b^	7 (26.9)	1 (3.8)	6 (23.1)	0.19
Post-OP Paresis X, *n* (%) ^b^	6 (23.1)	0	6 (23.1)	0.053
Post-OP Paresis XII, *n* (%) ^b^	3 (11.5)	1 (3.8)	2 (7.7)	1
Post-OP Dysarthria, *n* (%) ^b^	9 (34.6)	0	9 (34.6)	0.0038 **
Post-OP Dysphonia, *n* (%) ^b^	4 (15.4)	0	4 (15.4)	0.14
Post-OP abnormal cough, *n* (%)	4 (15.4)	0	4 (15.4)	0.14
Post-OP abnormal gag reflex, *n* (%)	3 (11.5)	0	3 (11.5)	0.26
Post-OP cough after swallow, *n* (%)	6 (23.1)	0	6 (23.1)	0.053
Post-OP wet voice after swallow, *n* (%)	3 (11.53)	0	3 (11.5)	0.26

a = Logistic regression analysis, b = Fisher’s exact test. * denotes significance with a threshold of p <0.05, ** denotes significance after correction for multiple testing (Bonferroni) with a threshold of *p* = 0.0042.

**Table 3:** Predictors for Post-Surgical Severe Dysphagia (FEDSS 4–6).

Clinical Parameters	All	Dysphagia FEDSS (1–3)	Severe Dysphagia FEDSS (4–6)	*p*-Value
*n* (%)	15	8 (53.3)	7 (46.7)	
Age, years (SD) ^a^	48 (±16)	49 (±18)	48 (±16)	0.64
Tumor size, mm (SD) ^a^	38 (±10)	39 (±9)	36 (±12)	0.67
Compression of brainstem, *n* (%) ^b^	12	6 (40.0)	6 (40.0)	1
Post-OP Paresis V, *n* (%) ^b^	3	0	3 (20.0)	0.06
Post-OP Paresis VII, *n* (%) ^b^	8	4 (26.7)	4 (26.7)	1
Post-OP Paresis IX, *n* (%) ^b^	6	0	6 (40)	<0.001 **
Post-OP Paresis XII, *n* (%) ^b^	2	0	2 (13.4)	0.18
Post-OP Paresis X, *n* (%) ^b^	6	1 (6.7)	5 (33.3)	0.034 *
Post-OP Dysarthria, *n* (%) ^b^	9	4 (26.7)	5 (33.3)	0.36
Post-OP Dysphonia, *n* (%) ^b^	4	1 (6.7)	3 (20.0)	0.26
Post-OP abnormal cough, *n* (%)	4	0	4 (26.7)	0.002 **
Post-OP abnormal gag reflex, *n* (%)	3	0	3 (20.0)	0.013 *
Post-OP cough after swallow, *n* (%)	6	1 (6.7)	5 (33.3)	0.002 **
Post-OP wet voice after swallow, *n* (%)	3	0	3 (20.0)	0.013 *

a = Logistic regression analysis, b = Fisher’s exact test. * denotes significance with a threshold of p <0.05,** denotes significance after correction for multiple testing (Bonferroni) with a threshold of *p* = 0.0042.
